# Estimation of Cyclic Shift with Delayed Correlation and Matched Filtering in Time Domain Cyclic-SLM for PAPR Reduction

**DOI:** 10.1155/2016/8506986

**Published:** 2016-09-26

**Authors:** Panca Dewi Pamungkasari, Yukitoshi Sanada

**Affiliations:** Department of Electronics and Electrical Engineering, Keio University, 3-14-1 Hiyoshi, Kohoku, Yokohama, Kanagawa 223-8522, Japan

## Abstract

Time domain cyclic-selective mapping (TDC-SLM) reduces the peak-to-average power ratio (PAPR) in OFDM systems while the amounts of cyclic shifts are required to recover the transmitted signal in a receiver. One of the critical issues of the SLM scheme is sending the side information (SI) which reduces the throughputs in wireless OFDM systems. The proposed scheme implements delayed correlation and matched filtering (DC-MF) to estimate the amounts of the cyclic shifts in the receiver. In the proposed scheme, the DC-MF is placed after the frequency domain equalization (FDE) to improve the accuracy of cyclic shift estimation. The accuracy rate of the propose scheme reaches 100% at *E*_*b*_/*N*_0_ = 5 dB and the bit error rate (BER) improves by 0.2 dB as compared with the conventional TDC-SLM. The BER performance of the proposed scheme is also better than that of the conventional TDC-SLM even though a nonlinear high power amplifier is assumed.

## 1. Introduction

Orthogonal frequency division multiplexing (OFDM) is multicarrier modulation which provides reliable high speed data rate because of its high spectral efficiency and its robustness against multipath fading channel. One of the significant problems of the OFDM signal is its high peak-to-average power ratio (PAPR) that requires wide range linearity in a power amplifier (PA). The high PAPR may drive a power amplifier into the saturation region, create interference among subcarriers, and corrupt the spectrum of the signal [1–3]. Some schemes for reducing PAPR have been available, for example, coding, filtering and clipping, and phase manipulation (selective mapping and partial transmit sequences).

Selective mapping (SLM) is one of the popular PAPR reduction schemes without signal distortion. SLM is a probabilistic scheme where signal candidates (SCs) are generated by multiplying the original signal sequence and the phase sequence. The SC with the lowest PAPR is chosen for transmission. In SLM, side information (SI) is needed at a receiver side to recover the transmitted signal. The SI is usually transmitted as a set of bits for every OFDM symbol and channel coding is required to protect it from a harsh channel. It involves the reduction of throughputs in wireless OFDM systems. Moreover, SLM has large computational complexity because it requires several inverse discrete Fourier transform (IDFT) operations and it makes the restrictions in implementation. A lower-complexity SLM scheme has also been proposed to solve this problem [4–15].

Many schemes have been proposed to exclude the SI [9–16]. The scheme in [9] makes the difference between the average energies of the extended and nonextended symbols to recover the SI at the receiver. As a consequence, higher order modulation symbols would influence the accuracy of SI detection. The scheme in [10] realizes semiblind SI detection in the SLM. However, this scheme requires embedding the SI in transmit symbols. In the scheme presented in [11], time domain cyclic-SLM with delayed correlation (DC) is applied to reduce a PAPR and to estimate the amount of a cyclic shift at the receiver without SI transmission. Nevertheless, there is a tradeoff between the amount of PAPR reduction and the BER. The method in [12] has been proposed to further reduce the PAPR of the abovementioned scheme. It uses matched filtering (MF) with a Barker sequence to estimate the amounts of cyclic shifts. One of the causes of the estimation error is multipath components. These components mislead the outputs of the DC-MF.

In this paper, time domain cyclic-SLM (TDC-SLM) without SI transmission is proposed. The proposed time domain cyclic-SLM (TDC-SLM) places the DC-MF after frequency domain equalization (FDE) to remove multipath components in a received signal. At a transmitter side, a transmit signal is generated by the summation of an original signal and signals with cyclic shifts. At a receiver side the amounts of the cyclic shifts are detected by using the DC-MF. In this proposed scheme, intervals between the cyclic shifts are designed so that the receiver can distinguish the cyclic shifts and multipath delays with the use of the MF. However, multipath components still deteriorate the accuracy rate of cyclic shift estimation since they generate additional peaks at the outputs of the DC-MF. By using the proposed scheme, the accuracy rate then improves and the bit error rate (BER) reduces as compared to that of the conventional TDC-SLM and DC-MF in [12].

The rest of this paper is organized as follows.  Section 1 contains the introduction.  Section 2 explains system models including the OFDM symbol structure, time domain cyclic-selective mapping, channel estimation and frequency domain equalization, and the proposed cyclic shift estimation scheme. In Section 3 the performance results of the proposed scheme are presented and finally Section 4 concludes this paper.

## 2. System Model

### 2.1. OFDM Symbol

The discrete OFDM signal *x*[*n*] in time domain can be written as

(1) $$\begin{array}{c} x\left[\;n\;\right]=\frac{1}{N}\overset{N-1}{\underset{k=0}{\sum }}X\left[\;k\;\right]exp\left(\;j\frac{2\pi nk}{N}\;\right),\;0\leq n\leq N-1, \end{array}$$

where *n* is the time index, *X*[*k*] is the data symbol on the *k*th subcarrier, *k* denotes the subcarrier index, and *N* is the number of the subcarriers. The OFDM signal can also be defined as a vector $x={\left[\begin{array}{cccc} x[0] & x[1] & \cdots & x[N-1] \end{array}\right]}^{T}$.

In order to mitigate the intersymbol interference, a guard interval (GI) is needed. The GI can be obtained by copying the last part of the OFDM signal and adding it to the beginning of the signal.

(2) $$\begin{array}{c} x_{f}\left[\;n\;\right]=\left\{\begin{array}{cc} x\left[n\right], & 0\leq n\leq N-1,\\ x\left[N+n\right], & -N_{GI}\leq n<0, \end{array}\right. \end{array}$$

where *N*_GI_ is the length of the GI.

### 2.2. Time Domain Cyclic-Selective Mapping

**Figure 1:**
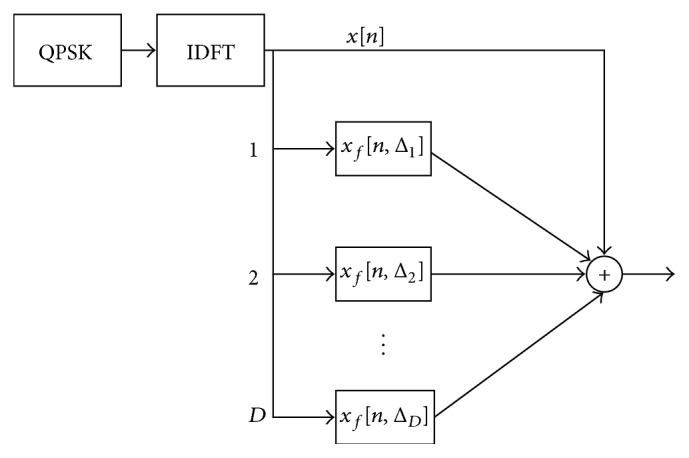
TDC-SLM with (*D* = 3) signal candidates.

In the TDC-SLM scheme, a signal on each branch is generated by applying a cyclic shift to the original signal. The block diagram of the TDC-SLM scheme is shown in Figure 1. The cyclically shifted signal in the TDC-SLM is given as

(3) xfn,Δd=xN−Δd+n,−NGI≤n≤Δd−1,xn−Δd,Δd≤n≤N−1,

where *x*[*n*] is the OFDM signal in the time domain at the time index of *n*, *N*_GI_ is the GI length, *x*_*f*_[*n*, Δ_*d*_] is the SC that is generated by cyclically shifting the OFDM signal by Δ_*d*_, Δ_*d*_ is the amount of the cyclic shift for the *d*th SC, and Δ_*d*_ ∈ {*Cδ*}, where *C* is an integer [11, 12]. The resolution of the cyclic shifts, *δ*, has to be large enough for accurate estimation of the cyclic shifts in a receiver.

The transmitter combines the SCs to the original signal in the time domain as follows:

(4) $$\begin{array}{c} s\left[\;n\;\right]=x_{f}\left[\;n\;\right]+\overset{D}{\underset{d=1}{\sum }}Q_{d}x_{f}\left[\;n,\Delta_{d}\;\right], \end{array}$$

where *s*[*n*] is the transmit signal, *D* is the number of branches, *Q*_*d*_ is the *d*th coefficient in the phase sequence, and *x*_*f*_[*n*] is the original signal with the GI. The same set of {Δ_*d*_} is applied over multiple symbols since the corresponding outputs of the DC-MF are averaged to improve the accuracy of cyclic shift estimation. Thus, the set of {Δ_*d*_} is selected so that the maximum PAPR over the symbols for averaging is minimized. Here, the PAPR is calculated for each OFDM symbol period.

(5) $$\begin{array}{c} PAPR={\left.\;10\;log_{10}\left[\;\frac{\max{\left|s\left[n\right]\right|}^{2}}{E\left\{{\left|s\left[n\right]\right|}^{2}\right\}}\;\right]\;\right|}_{0\leq n\leq N-1}, \end{array}$$

where *E*{·} denotes the expectation operation.

### 2.3. Channel Estimation and Frequency Domain Equalization

Frequency response and coarse symbol timing can be obtained by sending the preamble symbols at the beginning of the transmitted signal. The received preamble signal is given by

(6) $$\begin{array}{c} r_{p}\left[\;n\;\right]=\overset{N-1}{\underset{i=0}{\sum }}h\left[\;i\;\right]s_{p}\left[\;n-i\;\right]+w\left[\;n\;\right], \end{array}$$

where *s*_*p*_[*n*] and *r*_*p*_[*n*] are the *n*th transmitted and received preamble signals in the time domain, respectively. At the receiver, the preamble signal on the *k*th subcarrier is demodulated by taking a discrete Fourier transform (DFT) as

(7) $$\begin{array}{c} R_{p}\left[\;k\;\right]=\overset{N-1}{\underset{n=0}{\sum }}r_{p}\left[\;n\;\right]e^{-j2\pi nk/N}. \end{array}$$

The estimation of the channel frequency response on the *k*th subcarrier in the frequency domain, $\hat{H}[k]$, is given as follows:

(8) $$\begin{array}{c} \hat{H}\left[\;k\;\right]=\frac{R_{p}\left[k\right]}{S_{p}\left[k\right]}, \end{array}$$

where *S*_*p*_[*k*] is the transmitted preamble symbols on the *k*th subcarrier. Because of the TDC-SLM in the time domain, the channel frequency response needs to be modified during the data period. The superposition of the data sequence works like an artificial multipath on the channel response. To calculate the channel frequency response in the data period, the estimated channel response is converted to the impulse response in the delay domain as follows:

(9) $$\begin{array}{c} \hat{h}=IFFT\left(\;{\hat{H}}^{T}\;\right)={\left[\;\begin{array}{ccc} \hat{h}\left[0\right] & \cdots & \hat{h}\left[N-1\right] \end{array}\;\right]}^{T}, \end{array}$$

where $\hat{h}[i]$ is the *i*th impulse response of the channel, $\hat{H}={\left[\begin{array}{cccc} \hat{H}[1] & \hat{H}[2] & \cdots & \hat{H}[N] \end{array}\right]}^{T}$, and {·}^*T*^ denotes the transpose.

The coefficients of the minimum mean square error- (MMSE-) frequency domain equalization (FDE) for the *k*th subcarrier, *W*[*k*], are given by

(10) $$\begin{array}{c} W\left[\;k\;\right]={\hat{H}}^{*}\left[\;k\;\right]{\left(\;\hat{H}\left[\;k\;\right]{\hat{H}}^{*}\left[\;k\;\right]+\sigma^{2}\;\right)}^{-1}, \end{array}$$

where ${\hat{H}}^{*}[k]$ denotes the conjugate of the channel frequency response that is obtained from (8) and *σ* ^2^ is the variance of the noise estimated in the receiver. The demodulated signal on the *k*th subcarrier is then

(11) $$\begin{array}{c} \hat{R_{r}}\left[\;k\;\right]=W\left[\;k\;\right]R\left[\;k\;\right], \end{array}$$

where *R*[*k*] denotes the signal on the *k*th subcarrier at the receiver.

### 2.4. Cyclic Shift Estimation Scheme

The DC-MF is applied to the signal in the time domain after the MMSE-FDE to estimate the amounts of the cyclic shifts at the receiver. The received signal in the time domain can be written as

**Figure 2:**
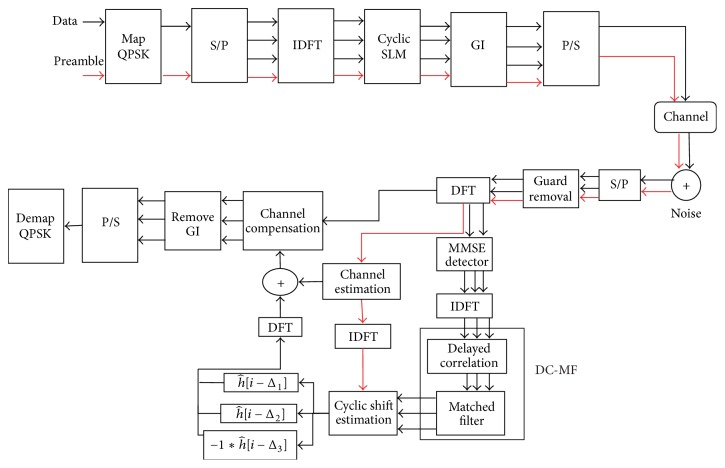
Block diagram of proposed scheme.

(12) rrn=1Nsn∑k=1NFn,kHk2Hk2+N0/Es+1N∑l≠nl=1N ∑k=1NSkFl,kHk2Hk2+N0/Es+∑k=1NWkHkHk2+N0/Es,

where *H*[*k*] is the channel frequency response, *S*[*k*] is the signal component, and *W*[*k*] is the Gaussian noise on the *k*th subcarrier. Furthermore, *r*_*r*_[*n*] is the *n*th received signal, *E*_*s*_/*N*_0_ is the signal-to-noise ratio per sample, $F_{j,n}=\exp\left[\left(2\pi i/N\right)jn\right]/\sqrt{N}$, *N* is the size of the DFT, and (·)^*∗*^ denotes conjugate. The DC-MF process consists of DC and MF as shown in Figure 2. In the transmitter side, the TDC-SLM generates several SCs by applying cyclic shifts to the original signal after the inverse DFT (IDFT) and generates the transmit signal through the summation of the original signal and the SCs. The DC-MF is utilized to estimate the amount of the cyclic shifts since they are required to recover the transmit signal.

**Figure 3:**
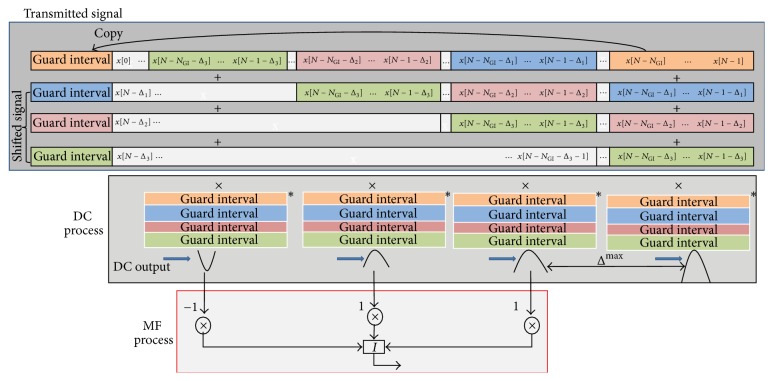
DC-MF process (*D* = 3).

Basically, the DC process multiplies the received signal in the time domain with the conjugate of the GI sequence. The largest peak appears when the last part of the OFDM symbol is multiplied with the conjugate of the GI. The output of the DC is put into the MF to estimate the set of the cyclic shifts, {Δ_*d*_}, by detecting the second largest peak output. The DC-MF processes with 3 branches are shown in Figure 3. The DC is defined as follows:
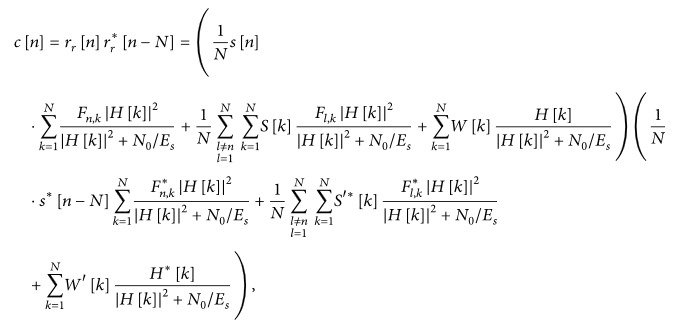
(13)where *s*[*n*] = *s* ^*∗*^[*n* − *N*] and *S* ^′*∗*^[*k*] and *W*′[*k*] are the signal and noise components on the *k*th subcarrier output from the delayed branch of the DC, respectively. After the summation, the outputs of the DC are averaged as follows:

(14) $$\begin{array}{c} C\left[\;n\;\right]=\frac{1}{V}\overset{V}{\underset{v=1}{\sum }}\overset{N_{GI}-1}{\underset{p=0}{\sum }}c\left[\;n-v\left(\;N+N_{GI}\;\right)-p\;\right], \end{array}$$

where *V* is the number of symbols used in the averaging process. The first peak is caused by the GI and is found by maximization as

(15) $$\begin{array}{c} n^{max}=\underset{n}{arg\;max}\left|\;C\left[\;n\;\right]\;\right|. \end{array}$$

In addition, the DC produces the correlation between the GI sequence and the received signal with the delay of ${\hat{\Delta }}_{d}$, that is, $r_{r}[n-{\hat{\Delta }}_{d}]$. The transmitted signal in the GI consists of the last part of the original OFDM signal, $\left[\begin{array}{ccc} x[N-N_{GI}] & \cdots & x[N] \end{array}\right]$, as well as the last part of the SC sequence, $\left[\begin{array}{ccc} x[N-N_{GI}-\Delta_{d}] & \cdots & x[N-1-\Delta_{d}] \end{array}\right]$. Hence, if ${\hat{\Delta }}_{d}=\Delta_{d}$, the DC outputs another peak as follows:

(16) $$\begin{array}{c} {\hat{c}}_{d}\left[\;n^{max}-{\hat{\Delta }}_{d}\;\right]=r_{r}\left[\;n^{max}-{\hat{\Delta }}_{d}\;\right]r_{r}^{*}\left[\;n^{max}-N\;\right], \end{array}$$

where ${\hat{\Delta }}_{d}$ is the candidate for the amount of the cyclic shift on the *d*th branch. Equation (16) can be rewritten as

(17) c^dnmax−Δ^d=∑ihisnmax−Δ^d−i+wnmax−Δ^d·∑i′h∗i′s∗nmax−N−i′+w∗nmax−N.

Therefore, the *d*th peak output of the DC is given as

(18) C^dnmax,Δ^d=1V∑v=1V∑p=0NGI−1c^dnmax−vN+NGI−Δ^d−p.

**Figure 4:**
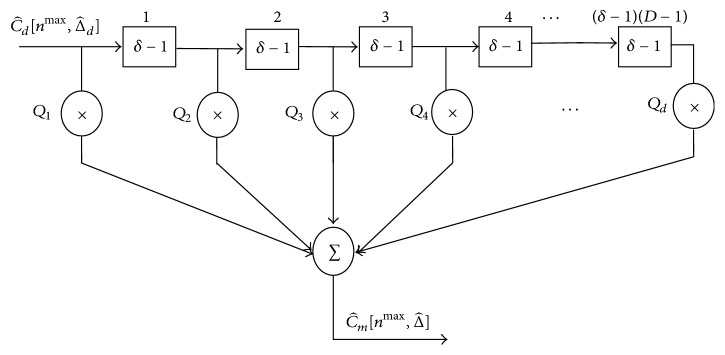
Matched filter.

The outputs of the DC are then passed to the MF to estimate the amounts of the cyclic shifts.  Figure 4 shows the structure of the MF. In order to reduce the number of the combinations of {Δ_*d*_}, here, the amounts of the cyclic shifts are selected from every *δ* samples as {Δ, Δ + *δ*,…, Δ + (*d* − 1)*δ*}. The structure of the MF has the delay line in which all the delays are set to (*δ* − 1). The output of the MF is expressed as follows:

(19) $$\begin{array}{c} {\hat{C}}_{m}\left[\;n^{max},\hat{\Delta }\;\right]=\overset{D}{\underset{d=1}{\sum }}Q_{d}{\hat{C}}_{d}\left[\;n^{max},\hat{\Delta }+\left(\;\delta -1\;\right)\left(\;d-1\;\right)\;\right], \end{array}$$

where ${\hat{C}}_{m}[n^{max},\hat{\Delta }]$ is the output of the MF.

The cyclic shift of the first branch, Δ, is estimated through maximization as

(20) $$\begin{array}{c} \Delta^{max}=\underset{\hat{\Delta }}{arg\;max}\left|\;{\hat{C}}_{m}\left[\;n^{max},\hat{\Delta }\;\right]\;\right|, \end{array}$$

where Δ^max^ is the estimated amount of the cyclic shift for the first branch, Δ. The channel impulse response in (9) is shifted by the estimated cyclic shifts and summed together with the original impulse response as follows:

(21) $$\begin{array}{c} {\hat{h}}_{d}\left[\;i\;\right]=\overset{D}{\underset{d=1}{\sum }}\left(\;\hat{h}\left[\;i\;\right]+\hat{h}\left[\;i-\left(\;\Delta +\left(\delta -1\right)\left(d-1\right)\;\right)\;\right]\;\right). \end{array}$$

The received signal in the frequency domain after channel compensation is given as follows:

(22) $$\begin{array}{c} \hat{X}\left[\;k\;\right]={\hat{H}}_{d}\left[\;k\;\right]R\left[\;k\;\right], \end{array}$$

where the channel response on the *k*th subcarrier in the data period is

(23) $$\begin{array}{c} {\hat{H}}_{d}\left[\;k\;\right]=\overset{N-1}{\underset{i=0}{\sum }}{\hat{h}}_{d}\left[\;i\;\right]\exp\left(\;-\frac{j2\pi ik}{N}\;\right). \end{array}$$

## 3. Numerical Results

### 3.1. Simulation Conditions

Table 1 shows the simulation parameters of the proposed scheme which are adopted from LTE parameters. The number of data subcarriers is 128 and each subcarrier is modulated with QPSK. The DFT size is 256 and the length of the GI is set to be 64 samples. The number of symbols for averaging is set to 1, 2, 4, or 8, and the range of the cyclic shift (Δ_*d*_) is limited from 60 to 124 with the resolution of every 4 samples (*δ*), which means 16 SCs. A convolutional code with a rate of 1/2 and constraint length of 7 with polynomial generator $\left[\begin{array}{cc} 171 & 133 \end{array}\right]$ is applied. The block interleaver with a size of 16 × 8 is applied. The number of branches is 3 and a Barker sequence with a length of 3 (*Q*_*d*_) is used as the phase sequence. A uniform delay profile with 6 paths is assumed as the channel model in computer simulation. As a nonlinear high power amplifier (HPA) Rapp's solid state power amplifier (SSPA) model with a knee factor of 3 is assumed. The input back-off (IBO) for the HPA is set to 0, 2, and 4 dB.

### 3.2. PAPR Reduction

**Figure 5:**
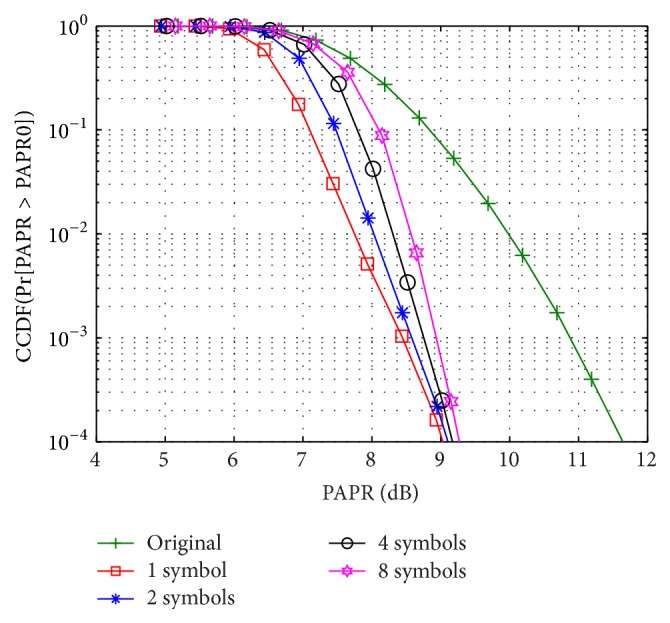
PAPR reduction.

The PAPR performance curves are evaluated in terms of complementary cumulative distribution functions (CCDF). The TDC-SLM assumes *D* = 3 branches with the cyclic shift resolution of *δ* = 4 and the number of the symbols for averaging is selected from 1, 2, 4, or 8. From Figure 5, it can be seen that, in comparison with the original signal, the amounts of PAPR reduction in the TDC-SLM for 1, 2, 4, or 8 symbols for averaging are 2.8 dB, 2.7 dB, 2.6 dB, and 2.5 dB, respectively, at the CCDF of 10^−4^. When the number of symbols for averaging is 1, each OFDM symbol has different set of the cyclic shifts and the best PAPR reduction is achieved. On the other hand, the smallest amount of PAPR reduction is realized where 8 OFDM symbols have the same set of the cyclic shifts in order to average the corresponding DC outputs. Nevertheless the difference in the amount of the PAPR reduction for 1 and 8 symbols for averaging is only 0.3 dB at a CCDF of 10^−4^.

### 3.3. Accuracy Rate and BER Performance

**Figure 6:**
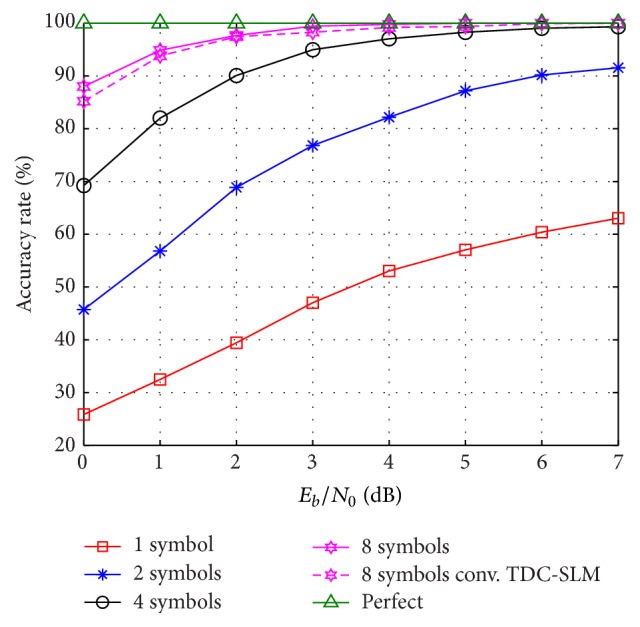
Accuracy rate on uniform delay profile channel.

The accuracy rate is the ratio of correct estimation in terms of the amounts of the cyclic shifts in the receiver side. The accuracy rate of the cyclic shift estimation is shown in Figure 6. Here, the uniform delay profile channel is assumed. In Figure 6, the accuracy rate of the proposed scheme for 1, 2, 4, or 8 symbols for averaging is around 57.04%, 87.80%, 98.28%, and 100%, respectively, at *E*_*b*_/*N*_0_ = 5 dB. The multipath channel affects the accuracy rate of the cyclic shift estimation. It is observed that the accuracy rates of the proposed scheme with 8 symbols for averaging are the highest as compared to the others. The accuracy rates of the proposed scheme and the conventional TDC-SLM with 8 symbols for averaging reach 100% at *E*_*b*_/*N*_0_ = 5 and *E*_*b*_/*N*_0_ = 7 dB, for 8 symbols for averaging, respectively. The accuracy rate performance is 2 dB better as compared to that of the conventional TDC-SLM scheme.

**Figure 7:**
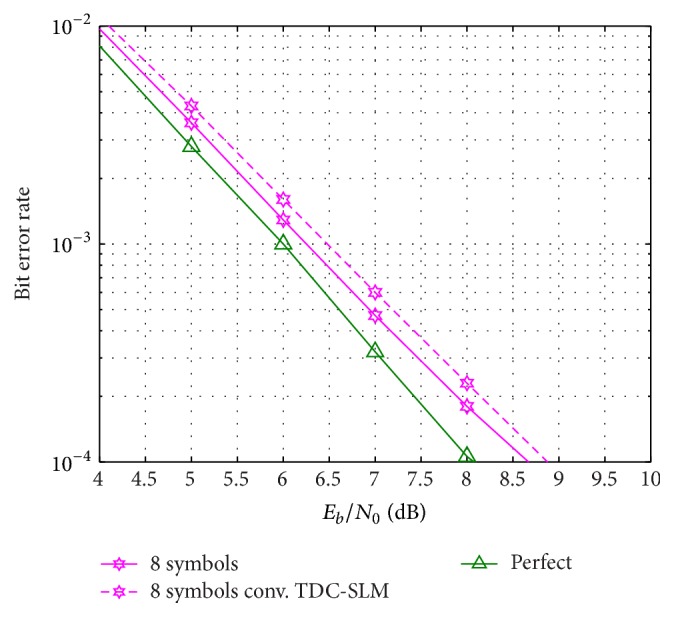
BER on uniform delay profile channel.

The accuracy rate affects the BER performance. The BER performance of the proposed scheme and the conventional TDC-SLM scheme with 8 symbols for averaging is presented in Figure 7 on the uniform delay profile channel. The difference between the BERs with perfect estimation and the proposed scheme with 8 symbols for averaging is 0.6 dB and it is 0.2 dB better than that of the conventional TDC-SLM scheme.

**Figure 8:**
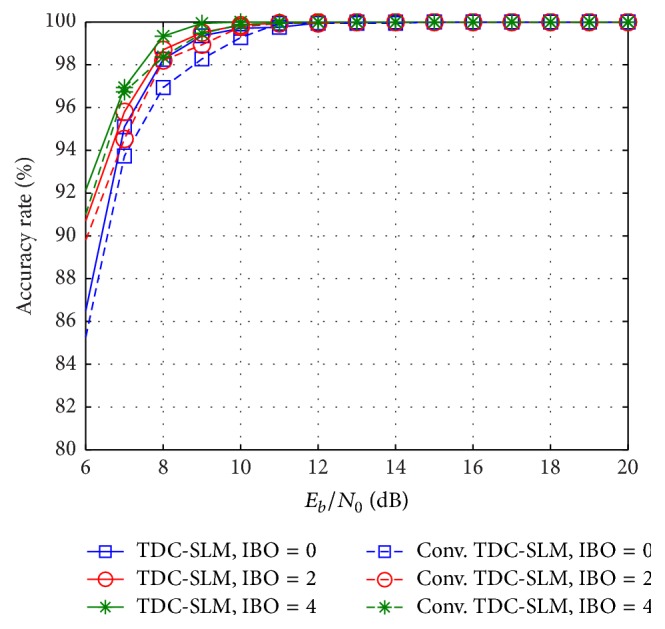
Accuracy rate with HPA on uniform delay profile channel.

The BER and the accuracy rate with the HPA are also evaluated.  Figure 8 presents the comparison of the accuracy rates between the proposed scheme and the conventional TDC-SLM. The number of the symbols for averaging is set to 8. The accuracy rate of the propose scheme reaches 100% when *E*_*b*_/*N*_0_ = 10 dB, 11 dB, and 13 dB for IBO of 4 dB, 2 dB, and 0 dB, respectively. On the other hand, the accuracy rate of the conventional TDC-SLM realizes 100% at *E*_*b*_/*N*_0_ = 11 dB, 12 dB, and 14 dB for IBO of 4 dB, 2 dB, and 0 dB, respectively. It shows that the accuracy rate of the proposed scheme improves when the DC-MF is placed after FDE-MMSE even though the nonlinearity of the HPA is assumed. The accuracy rate performance is 1 dB better as compared to that of the conventional TDC-SLM scheme.

**Figure 9:**
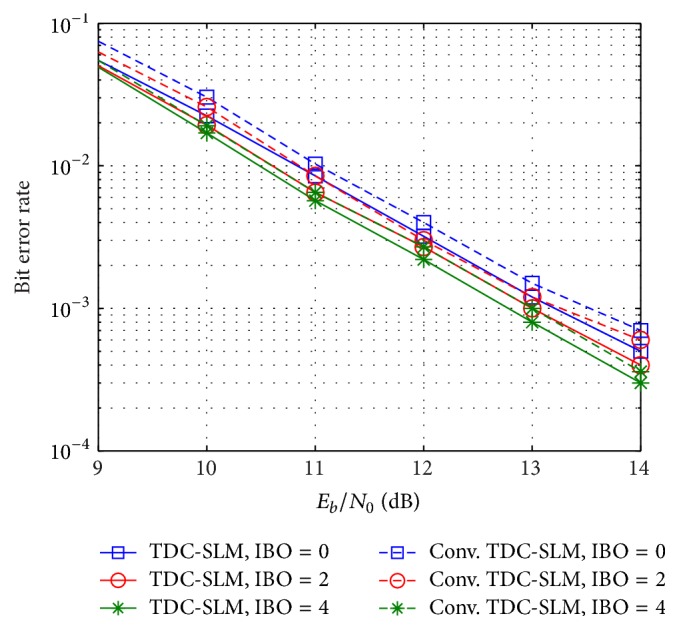
BER with HPA on uniform delay profile channel.

The BERs for the different values of the IBO are also evaluated as depicted in Figure 9. In the conventional TDC-SLM, the required *E*_*b*_/*N*_0_ values at a BER of 10^−3^ are 13 dB, 13.3 dB, and 13.8 dB, for IBO of 4 dB, 2 dB, and 0 dB, respectively. On the other hand, with the proposed scheme, it is 12.8 dB, 13.1 dB, and 13.3 dB for IBO of 4 dB, 2 dB, and 0 dB, respectively. It is observed that the BER improves by increasing the IBO. The BER differences between the conventional TDC-SLM and the proposed scheme are 0.4 dB, 0.3 dB, and 0.4 dB at a BER of 10^−3^ for IBO of 4 dB, 2 dB, and 0 dB, respectively. The proposed scheme is proven to improve the accuracy rate and the BER performance as they approach the values with perfect estimation.

## 4. Conclusions

In this paper, the SI detection scheme for the TDC-SLM has been proposed. The DC-MF is implemented after the MMSE detection to remove the effect of the multipath channel. The amount of PAPR reduction with the TDC-SLM is around 2.5 dB as compared with that of the original signal for 8 symbols for averaging when the resolution of the cyclic shift is *δ* = 4 samples. The accuracy rate of the proposed scheme reaches 100% at *E*_*b*_/*N*_0_ = 5 dB and the BER difference with 8 symbols for averaging is around 0.6 dB as compared to that with the perfect estimation of the SI. The BER is 0.2 dB better than that of the conventional TDC-SLM scheme. Under the nonlinearity of the HPA, the proposed scheme still improves the BER performance by around 0.4 dB at a BER of 10^−3^ for IBO of 4 dB, 2 dB, and 0 dB.

## Figures and Tables

**Table 1:** Simulation conditions.

Modulation	QPSK/OFDM
DFT size	256
Number of data subcarriers	128
Guard interval	64
Number of symbols for averaging	1, 2, 4, 8 symbols
Cyclic shift (Δ_*d*_)	From 60 until 124
Resolution of cyclic shift (δ)	4
Coding scheme	Convolutional coding
Interleaver	Matrix: 16 × 8
Decoding scheme	Soft decision Viterbi
Constraint length	7
Coding rate	1/2
Polynomial generator	[171 133]
Number of branches	3
Channel	Uniform (6 paths)
Knee factor power amplifier (*p*)	3
Input backoff (IBO)	0, 2, 4 dB

## References

[1] Rahmatallah Y., Mohan S. (2013). Peak-to-average power ratio reduction in ofdm systems: a survey and taxonomy. *IEEE Communications Surveys and Tutorials*.

[2] Bae K., Shin C., Powers E. J. (2013). Performance analysis of OFDM systems with selected mapping in the presence of nonlinearity. *IEEE Transactions on Wireless Communications*.

[3] Jiang T., Wu Y. (2008). An overview: peak-to-average power ratio reduction techniques for OFDM signals. *IEEE Transactions on Broadcasting*.

[4] Wang C.-L., Ouyang Y. (2005). Low-complexity selected mapping schemes for peak-to-average power ratio reduction in OFDM systems. *IEEE Transactions on Signal Processing*.

[5] Yang L., Soo K. K., Siu Y. M., Li S. Q. (2008). A low complexity selected mapping scheme by use of time domain sequence superposition technique for PAPR reduction in OFDM system. *IEEE Transactions on Broadcasting*.

[6] Wang J. S., Hwang S. H., Kim C. J., Kim Y. H. Time-domain signal combining with cyclic delay and phase shift for PAPR reduction in OFDM systems.

[7] Heo S.-J., Noh H.-S., No J.-S., Shin D.-J. (2007). A modified SLM scheme with low complexity for PAPR reduction of OFDM systems. *IEEE Transactions on Broadcasting*.

[8] Jeon H.-B., No J.-S., Shin D.-J. (2011). A low-complexity SLM scheme using additive mapping sequences for PAPR reduction of OFDM signals. *IEEE Transactions on Broadcasting*.

[9] Le Goff S. Y., Al-Samahi S. S., Khoo B. K., Tsimenidis C. C., Sharif B. S. (2009). Selected mapping without side information for PAPR reduction in OFDM. *IEEE Transactions on Wireless Communications*.

[10] Badran E. F., El-Helw A. M. (2011). A novel semi-blind selected mapping technique for PAPR reduction in OFDM. *IEEE Signal Processing Letters*.

[11] Pamungkasari P. D., Sanada Y. PAPR reduction using cyclic-selective mapping with delayed correlation in time domain.

[12] Pamungkasari P. D., Sanada Y. Time domain cyclic-selective mapping for PAPR reduction using delayed correlation with matched filter in OFDM system.

[13] Pamungkasari P. D., Sanada Y. (2015). Cyclic shift estimation with delayed correlation and matched filtering in cyclic-SLM PAPR reduction. *IEICE Technical Report*.

[14] Park J., Hong E., Har D. (2011). Low complexity data decoding for SLM-based OFDM systems without side information. *IEEE Communications Letters*.

[15] Hong E., Kim H., Yang K., Har D. S. (2013). Pilot-aided side information detection in SLM-based OFDM systems. *IEEE Transactions on Wireless Communications*.

[16] Eom S.-S., Nam H., Ko Y.-C. (2012). Low-complexity PAPR reduction scheme without side information for OFDM systems. *IEEE Transactions on Signal Processing*.

